# Prediction of Kerf Width and Surface Roughness of Al6351 Based Composite in Wire-Cut Electric Discharge Machining Using Mathematical Modelling

**DOI:** 10.3390/ma15031102

**Published:** 2022-01-30

**Authors:** Hariharan Sree Ram, Marimuthu Uthayakumar, Shanmugam Suresh Kumar, Sundaresan Thirumalai Kumaran, Brian Azzopardi, Kinga Korniejenko

**Affiliations:** 1 Faculty of Mechanical Engineering, Kalasalingam Academy of Research and Education, Krishnankoil 626126, India; hsreeram@amaljyothi.ac.in (H.S.R.); sureshme48@gmail.com (S.S.K.); thirumalaikumaran@yahoo.com (S.T.K.); 2 MCAST Energy Research Group, Malta College of Arts, Science and Technology, 2001 Paola, Malta; brian.azzopardi@ieee.org; 3 Department of Systems and Control Engineering, Faculty of Engineering, The University of Malta, 2080 Msida, Malta; 4 Foundation for Innovation and Research, 1129 Valletta, Malta; 5 Faculty of Materials Engineering and Physics, Cracow University of Technology, 31-155 Cracow, Poland; kinga.korniejenko@pk.edu.pl

**Keywords:** electric discharge machining (EDM), composite properties, Al6061 composite, mathematical modelling, surface roughness (SR), kerf width

## Abstract

The machining of composite materials has been an area of intense research for the past couple of decades due to its wide range of applications, from automobiles to air crafts or from boats to nuclear systems. Non-conventional machining, especially electric discharge machining (EDM), is found to be a good machining option for meeting the required outputs. To overcome the challenges of machining complex shapes, wire electric discharge machining (WEDM) was developed. Al6351 composites was observed to be extensively used in nuclear applications. Therefore, identifying the kerf width and surface roughness are important criteria for the dimensional accuracy of the final product. The present work aims at predicting the behavior of the two major machining parameters which are kerf width and surface roughness of Al6351 composites in wire EDM by creating a mathematical model using ANOVA for different combinations of the reinforcements and comparing the variations in the coefficients for different combinations of reinforcements. The developed model has been validated by conducting similar set of experiments in Al6351-5% SiC-1% B_4_C hybrid composite. From the work, it was identified that pulse on time and current are the major contributing factor for kerf width and wire feed rate was observed to be contributing to the surface roughness. The validation results show an average variation of 8.17% for kerf width and 11.27% for surface roughness. The work can be successfully utilized for prediction of the kerf width and surface roughness of the composites manufactured with Al6351 as the base matrix material.

## 1. Introduction

A challenge faced by present-day industry is to manufacture lightweight and durable materials at low cost to markets. Growing competitions and variations in customer requirements have led to development of quicker and newer products that meet customer requirements and provide customer delight in the marketplace. This has led to intense research in the area of composite materials which are easy to manufacture, having higher strength-to-weight ratio and development and modifications can be carried out at a faster rate than conventional materials. The identification of lighter materials that can bear increased loads is an area of material research in the present scenario. But the machining of these composites poses a huge challenge to be faced by researchers and identifying the behavior of a newly developed composite for a specific machining process is a big challenge for present-day researchers. The conventional machining process was observed to be time-consuming and costly for the machining of these materials due to frequent tool change leading to increased non-productive time. This led to the identification of non-conventional machining processes where the tool and workpiece will not have physical contact. Wire electric discharge machining (WEDM) was identified to be the best option for the machining of these difficult-to-machine composites. This work aims at providing an insight on the area of predicting the outputs of machining of Al6351 based composites using WEDM based on the hardness of the material using mathematical modelling and developing equation for the prediction of kerf width and surface roughness for a new material using functional equations developed. The equations developed were validated by carrying out experiments and comparing the experimental results with the model values.

Suresh Kumar et al. [1] used grey relational analysis to optimize the parameters such as peak current, pulse-on-time, and wire feed rate for the output parameters of kerf width and surface roughness for varying percentages of B_4_C in Al6351-SiC composite. The work identified that kerf width was increasing with increase in the pulse on time as well as increase in percentage of B_4_C in the composite. The pulse duration also showed a similar effect in surface roughness. Lal et al. [2] studied the significant machining process parameters for Al7075–Al_2_O_3_–SiC composites. The work identified that the pulse-on-time is the most significant factor for kerf width and surface roughness of the composite. Muniappan et al. [3] carried out the experiments of Al6061 hybrid composites with SiC and graphite as the reinforcements considering current, pulse-on-time, pulse-off-time, gap set voltage, wire drum speed, and wire tension as the input parameters for optimizing the kerf width and surface roughness. He concluded that pulse-on-current and gap set voltage played a significant role for the output parameters when compared to the other factors which were considered for the work.

Karabulut et al. [4] carried out experimental investigation of wire EDM in Al6061 with 15% B_4_C reinforcement. The work was carried out with three input parameters for investigation of surface roughness (R_a_) of the material. The input parameters considered were current, gap voltage, and wire tension. It was found that the current was the major contributing parameter for surface roughness. The lower current improves the surface roughness. The lower wire tension was also found to improve the surface roughness. The gap voltage was found to be the least contributing factor for surface roughness in the Al6061–15%B_4_C composite. Ramesh et al. [5] carried out experiments on Al6061–SiC composites and Al6061–SiC–B_4_C hybrid composite machining in EDM and wire EDM. The experiment was carried out with different percentages of SiC and B_4_C in the composite matrix. The three combinations which was prepared for the experiments were, Al6061–3% SiC_p_, Al6061–3% SiC_p_–3% B_4_C_p,_ and Al6061–7% SiC_p_–3% B_4_C_p_.The input parameters considered were current, pulse-on-time, pulse-off time, and gap voltage. The material removal rate and the surface roughness were the output parameters studied in this work. It was found from the analysis that the surface roughness increases with increase in the presence of SiC or B_4_C in the composite due to its higher hardness. For Al6061–3% SiC, the voltage was found to be the contributing factor for surface roughness whereas pulse-on-time contributes for the material removal rate. For Al6061–3% SiC_p_–3% B_4_C_p_ both the parameters were dominated by pulse on time per the ANOVA method. For Al6061–7% SiCp–3% B_4_C_p_, it was identified that the voltage is the predominant factor in controlling the output parameters.

Das et al. [6] carried out experiments for the optimization in the machining of Al6061–SiC–B_4_C nano particle hybrid composite. The base matrix was reinforced with 1.5% SiC and 1.5% B_4_C in this work. The optimization was carried out using response surface methodology. Ekici et al. [7] carried out experiments on Al–B_4_C composites for optimizing the material removal rate and surface roughness. The input parameters considered were wire tension, reinforcement percentage, wire diameter, pulse-on-time, and pulse-off-time. The Taguchi method was employed for the optimization of the parameters. It can be observed that the work related to wire EDM in composites were scarce and there was a huge scope for research in the segment. It was identified that the wire tension had no significant contribution on both the output parameters. The pulse-on-time was the major contributor to the surface roughness and wire speed was found as major contributor for the Material Removal Rate (MRR). The work also identified that the surface roughness increased with the increased percentage of reinforcements. Ma et al. [8] worked on a newer algorithm which was wolf pack algorithm to identify the optimum solution for wire EDM machining of Al–SiC composites after implementing the Gaussian regression. The work considered pulse duration, pulse interval, water pressure, and wire tension as the input parameters for the identification of variation in the output parameters considered, which were material removal rate and 3D surface characteristics. It was pointed out that the 3D surface characteristics can provide more clarity in the surface roughness when compared to the conventional 2D evaluation of surface roughness. After employing the Taguchi method, it was identified that the pulse duration or pulse-on-time was the most influential factor for all the output parameters. The conclusions were verified using Gaussian progress regression (GPR), linear regression model (LRM), and back propagation neural network (BPNN). It was identified that GPR possess the best and stable option for obtaining a predictable model. The wolf pack algorithm was used for identifying the optimum values of the input parameters for better output parameters. The work concluded that the GPR–Wolf Pack Algorithm (WPA) combination can provide an optimum values of input parameters more accurately when compared with other methodologies.

Garg et al. [9] worked on the optimization of the wire EDM machining of the Al/ZrO_2_ metal matrix composite. He used different optimization technique to identify the optimum values of the input parameters. The box cox method was employed for identifying the relation of variation of the output parameters with respect to the input parameters. It was identified that the maximum MRR that can be achieved in the optimized system is 25.375 mm^3^/min with spark gap of 0.017 mm which makes the system suitable for machining of the concerned composite material. It was identified that the wire EDM can be effectively used for the machining with good surface finish and material removal rate for Al/ZrO_2_ metal matrix composite. Shandilya et al. [10] worked on the Al6061–SiC metal matrix composite wire EDM optimization, using Response Surface Methodology (RSM) and Artificial Neural Network (ANN) methods. The parameters considered were servo voltage, pulse-on-time, pulse-off-time, and wire feed rate. The output parameters considered were material removal rate and kerf width. A comparison of the RSM model and ANN method was carried out to identify the effectiveness of both the methods. The work concluded that servo voltage and pulse-off-time contributed maximum to the parameters. A low servo voltage with medium pulse-on-time, low pulse-off-time, and low wire feed rate can provide maximum MRR and minimum kerf width. It was also found that artificial neural network (ANN) provided better results when compared to the RSM method. It was also mentioned that the ANN system is comparatively complicated and time-consuming. Saravanan et al. [11] experimentally identified the critical parameters that will affect the material removal rate and surface roughness of AA6063–TiC composite materials for different percentage of reinforcement of TiC. The major factor affecting the output parameters were found to be the percentage of reinforcement followed by the pulse-on-time. Peak current was found to be the least contributing factor for the considered parameters. Sureshkumar et al. [12] carried out an experimental study on the machining of Al6351–iC–B_4_C hybrid composite using die sinking EDM with copper as the tool electrode. The work identified that the material properties of the composites enhanced with the addition of B_4_C particles in the composites. The addition of B_4_C increased the heat-affected zone leading to increased white layer formation. The crater size of the machined surface also found to increase due to the presence of B_4_C particles. Sureshkumar et al. [13] conducted experiment on EDM for Al6351–5% SiC–5% B_4_C hybrid composite with the input parameters as current, pulse-on-time, duty factor, and gap voltage to evaluate the electrode wear rate, surface roughness, and power consumption. The work identified that the current is the major contributing factor for all the evaluated output parameters. Selva Babu et al. [14] carried out experiments on Al6061 alloy in wire EDM to optimize the surface roughness and material removal rate. It was identified that the current is the major parameter contributing for both the surface roughness and material removal rate followed by the pulse-on-time. Reddy et al. [15] worked on the optimization of the wire EDM of aluminium composite reinforced with silicon. The work targeted to reduce the energy consumption and improve the machining parameters. Two methods were adopted for the optimization viz. graph theory and utility concept (GTUC) and teaching learning-based optimization (TLBO) algorithm. It was identified that the TLBO produced better results when compared to the GTUC method. The energy consumption was reduced by 40% and kerf width by 2.8%. But the MRR was found to reduce by 43% which was aimed to be maximum. The surface roughness was also improved when the TLBO algorithm was used. Modrak et al. [16] carried out a study on Al–Mg based composites with MoS2 as the reinforcements. The work was carried out using two methods, viz., Non-Dominated Sorting Generic Algorithm-II (NSGA-II) and Multi-Objective Particle Swarm Optimization (MOPSO) for optimization. The work identified that MOPSO provides a better and quicker optimization when compared to NSGA-II and the optimized results were validated by carrying out experiments which were showing very close results to the evaluated results. Perla et al. [17] carried out investigations on wire EDM for nano hybrid aluminium composite with graphene and carbon nanotube added to Al6082. The investigations were carried out for optimizing surface roughness, material removal rate, and kerf width. The input parameters considered were pulse current, pulse-on-time, gap voltage, and wire feed rate. The work identified that the hardness and the electrical conductivity of the composite improved upon addition of the reinforcements. The pulse-on-time and the gap voltage were identified to be the contributing parameters for all the output parameters. Palanisamy et al. [18] worked on the aluminium graphene nano platelets (GNP) composites for identification of its machining parameters in wire EDM using grey relational analysis and developed an Adaptive Network-based Fuzzy Inference System (ANFIS) model to predict the behaviour. The output parameters of material removal rate, wire wear rate, and kerf width were evaluated for the input parameters of pulse-on-time, pulse-off-time, servo voltage, flushing pressure, and wire feed rate. The work identified that the methodology adopted can be successfully utilised for the evaluation process of the Al–GNP composites. Sreeraj et al. [19] worked on the wire EDM of Al6351–10% rutile composite for machining of microchannels in the electronic circuits. The work aimed at optimization of the kerf width, kerf depth, and the surface roughness using the Taguchi method and GRA with PCA technique to develop a hybrid evaluation model for the optimization of the output parameters. The input parameters considered in the work were current, voltage, and wire feed rate. The work identified current as the major contributor for the output parameters. The model was able to predict the optimized input parameters with close values to the experimental results. Mandal et al. [20] conducted wire EDM experiments on Al6061 alloy with the input parameters as pulse-on-time, pulse-off-time, voltage, and wire tension. The aim of the work was to minimize kerf width as well as surface roughness. Current which was identified to be a major contributor was not considered for this study. The work concluded that pulse-on-time is the major contributor for both kerf width and surface roughness. The work concluded that the reduced pulse-on-time can reduce both kerf width and surface roughness. Karmakar and Maji [21] carried out a comprehensive study on the research carried out on the recent developments in wire EDM. The study identified that most of the works are concentrated on aluminum composites with silicon carbide, boron carbide, aluminum oxide, etc. The study identified a lot of areas for the future work in the area of wire EDM on different composites of nickel, titanium, and related materials. The metallographic study of the machined surfaces was also found to be limited. Muniappan et al. [22] worked on machining of different combinations of Al6061–SiC–graphite hybrid composite using wire EDM. The work considered pulse-on-time, pulse-off-time, pulse current, gap voltage, wire drum speed, and wire tension as the input parameters. The output parameter considered was kerf width. The work identified that pulse-on-time is the dominating factor for the variation in kerf width for the combinations. Balasubramanian et al. [23] worked on the wire EDM machining of AA6063–SiC composite with different compositions of SiC. The experiments were carried out with 0, 5, 10, and 15% SiC added to the matrix material. The variations of kerf width and surface roughness based on the input parameters of peak current, pulse-on-time, and pulse-off-time were considered for the study. The work identified that pulse-on-time is the significant factor for surface roughness whereas pulse-off-time plays a significant role in the kerf width control. Paneer Selvam and Ranjith Kumar [24] worked on the optimization of wire EDM machining of Hastalloy C-276 using genetic algorithm. The input parameters of current, pulse-on-time, pulse-off-time, gap voltage, wire speed, and machine speed were considered for this work. The output parameters evaluated were machining time, surface roughness, and kerf width. The work identified that genetic algorithm can be employed for the successful prediction of the output parameters of the wire EDM but it can be understood based on the methodology employed that the development of the codes is complicated. Modi et al. [25] carried out the study on the machining of Al–15% SiC metal matrix composite (MMC) in wire EDM. The study aimed at optimizing the kerf width. MRR and surface roughness in relation to the machine feed rate. It was identified that the reduced machine feed rate leads to more kerf width and MRR. But it improves the surface finish. Amruth Babu and Gurupavan [26] carried out experimental study on the wire EDM machining of Al6061 composite with the varying reinforcement percentage of SiC. The input parameters considered for this work were current, pulse-on-time, pulse-off-time, and wire feed rate. The work concluded that the surface roughness reduced with the addition of SiC in the base alloy. The work also identified that the increase in the percentage of SiC led to lower surface finish. Ishfaq et al. [27] targeted his work on wire EDM machining of Al6061–7.5% SiC composite. The work aimed at optimizing the kerf width, surface roughness, and cutting rate of the based on the input parameters of current, voltage, and pulse-on-time. The dominating factor that controls the surface roughness was identified to be the voltage. Current was identified to be the dominating factor for kerf width and the pulse-on-time was the dominating factor in cutting rate. Better surface finish was achieved with lower current and voltage. The work also identified the presence of narrow craters in the machined surface at lower voltage and current. Kashif Ishfaq et al. [28] carried out experimental study for the machining of Al6061–7.5% SiC composite using wire EDM. The work highlighted upon the challenges in the machining of Al6061 based composites. The work calculated the errors due to the wire vibrations and lag. The work evaluated the corner variations and errors in cutting orientations. These variations were identified to be due to the presence of SiC as the reinforcement element in the composite. The work also identified the involvement of variations of current, pulse-on-time, and voltage on the considered output parameters. Marafona and Araujo [29] conducted extensive work on the effect of the workpiece hardness for the EDM process. It was identified that the workpiece hardness and related parameters have significant influence in the surface roughness and MRR of the EDM process. The work aimed at developing a model based on the input data for steel and identified that the derived model is able to predict the values very close to the experimental values.

Most of the work related to the wire EDM was found to optimize the surface roughness and the kerf width as these two were found to be critical factors of the component. The works related to Al6351 based composites were found to be very less due to the availability of the material for the base matrix. The composite has been extensively used in nuclear applications which requires machining after making the composite using stir casting. It was identified that there were no major models being created for Al6351 based composites for predicting the kerf width and surface roughness A mathematical model can help the researchers and industrial works related to nuclear industry for optimizing the major input parameters for obtaining the required output. Therefore, the present work aims at creating a mathematical model for predicting these major contributing parameters based on the hardness of the material. The data for the work have been taken from the work carried out by Sureshkumar et al. [1] where Al6351 with SiC and B_4_C as reinforcements had been used for the study. Since there are only three variants available in the literature, three more materials were developed and experimented for obtaining a good regression model. The objectives of the present work are:

- To obtain the optimum mathematical model for Al6351–5% SiC metal matrix composite with different combinations of B_4_C;
- To identify the pattern of variation of coefficients of different input parameters used in the mathematical model based on the hardness of each material;
- To create an equation for each coefficient, thereby predicting the mathematical model for a new combination, once the hardness of the material is obtained; and
- To validate the result by carrying out experiments and comparing the model with the experimental results.

## 2. Materials and Methods

**Figure 1:**
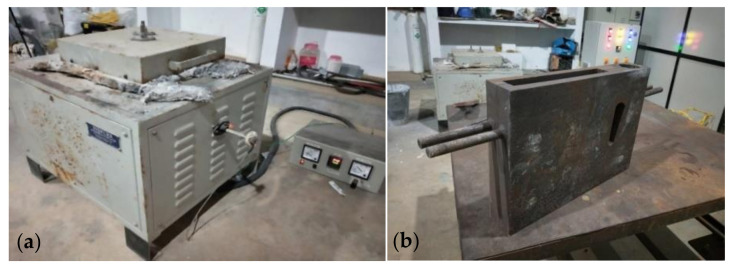
(**a**) Stir casting furnace set up; and (**b**) casting die.

The work is based on the publication by Sureshkumar [1] where three different combinations of Al6351–SiC–B_4_C composites were manufactured and tested for kerf width and surface roughness. Since three results will not provide a satisfactory regression model, four more specimens were developed, three for the additional values in the regression model and one for the validation of the developed model. The stir casting was adopted for the manufacturing of the composites. Figure 1 shows the different stages of manufacturing of the specimen by stir casting. The methodology adopted were as follows:

- Initially the casting die is preheated to 400 °C;
- Al6351 cylindrical rod which is cut to length of 100 mm was added into the crucible and kept in the furnace;
- The base material was heated to the temperature of 850 °C for taking it above the liquidous state;
- The molten metal is stirred using a stirrer at 700 rpm and allowed to cool down slowly;
- The reinforcements (SiC and B_4_C) were slowly added to the molten metal without stopping the stirring action;
- 2% magnesium is added to the molten composite to improve its wettability;
- The molten mixture is poured into the preheated rectangular die of size 200 mm × 150 mm × 30 mm to obtain the final composite; and
- The poured composite was allowed to cool down in the die to obtain the final specimen in the solid condition.

**Figure 2:**
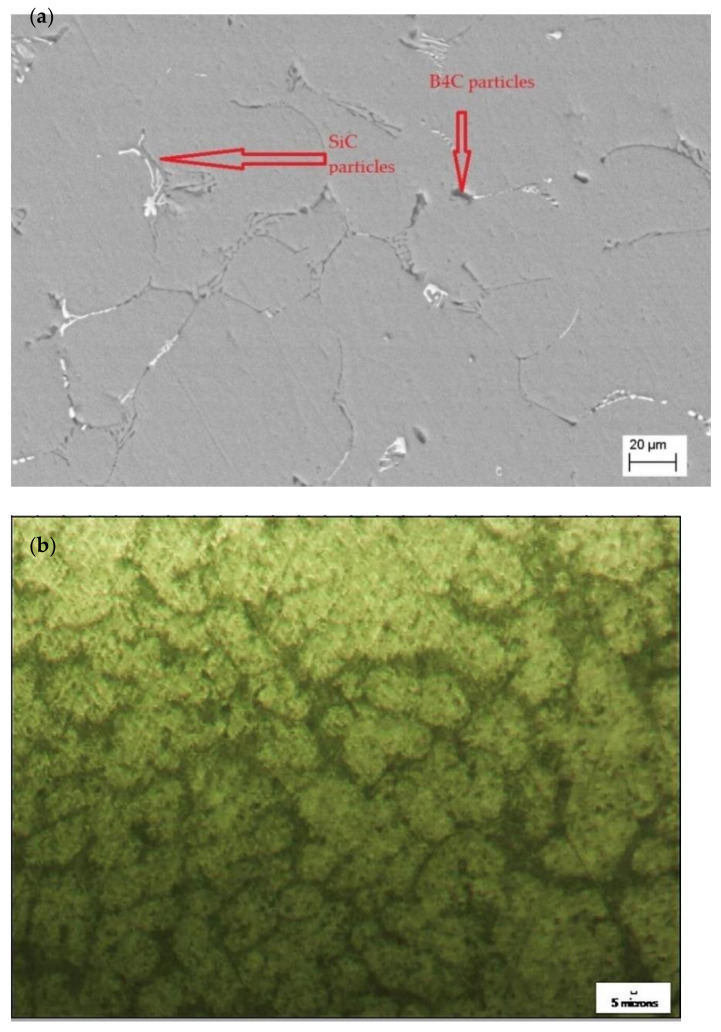

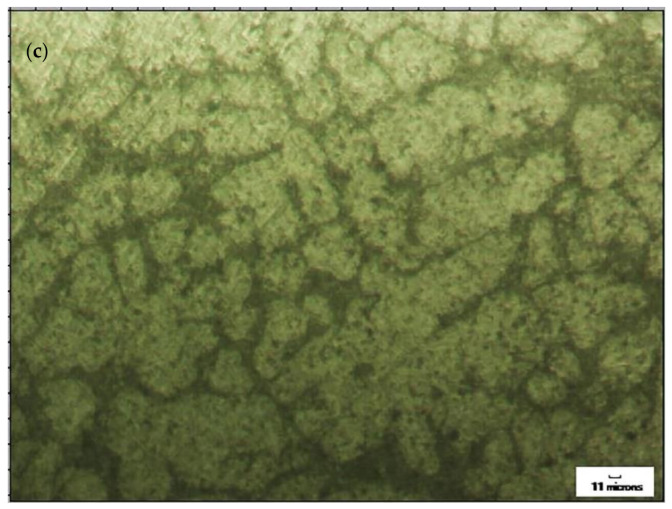
Figures for displaying the microstructure of the composite: (**a**) SEM image of the specimen; (**b**) microscopic image with 100× magnification; and (**c**) microscopic image with 200× magnification.

**Figure 3:**
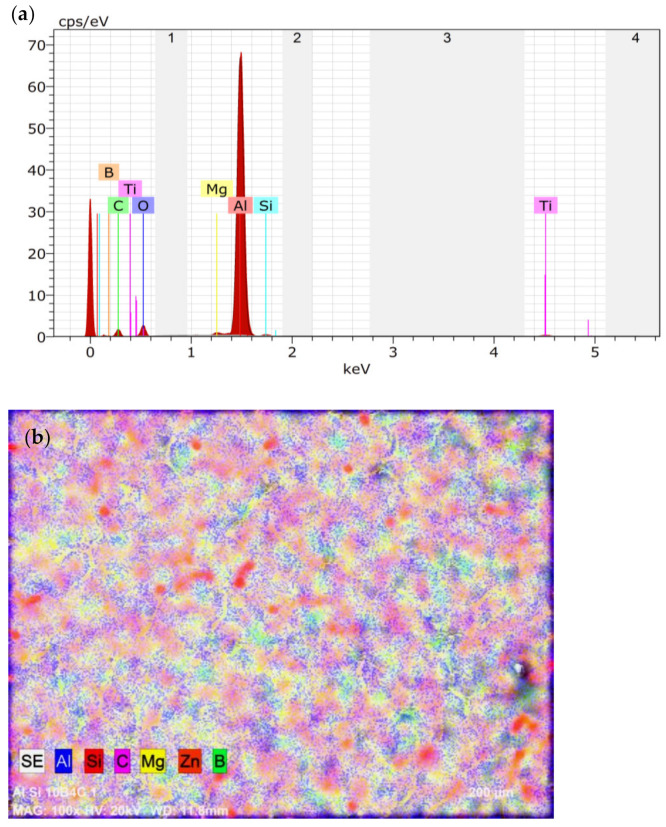
Analysis provided for the fabricated composite: (**a**) EDAX and (**b**) elemental mapping.

Scanning electron microscope as well as optical microscopic analysis was carried out for the specimen to identify the proper distribution of the reinforcements in the base matrix and the grain boundaries. The optical microscope employed for the analysis was QS–17AT manufactured by M/s QS Metrology (New Delhi, India). The magnification available in the microscope were 100, 200 and 400× (10× at the eyepiece and 10, 20, and 40× at the achromatic objective). The details of the images are provided in Figure 2a–c. The Energy Dispersive X-ray Analysis (EDAX) to identify the presence of elements of the reinforcements were also carried out which is provided in Figure 3a,b.

The sample preparation for the microscopic analysis consists of the following steps:

- The sample to be tested is first polished manually using a series of emery paper 1/0, 2/0, 3/0, and 4/0;
- The hand-polished specimen is repolished by using mechanically rotating wheel covered with polishing cloth and simultaneously, alumina powder mixed in water was poured on the wheel area where polishing was carried out;
- For mirror-type surface finish, diamond paste was used on the clean surface;
- The sample was cleaned using water and Kellars etchant, which is a mixture of nitric, hydrochloric, and hydrofluoric acids, is applied on the surface for revealing of microstructure; and
- The sample is dried using a hand drier and carefully preserved for the microstructure analysis without any contact with the polished surface.

The microscopic images of the sample show uniform distribution of the reinforcements in the base matrix and the deposition of the reinforcements in the grain boundaries can be observed. The reinforcements in the grain boundaries improve the mechanical properties of the composite.

The EDAX analysis for identifying the constituents present in the composites were also carried out. It can be observed that the presence of all the reinforcement elements can be clearly identified in the EDAX analysis.

The composite was machined in the wire EDM machine located at M/s A1 Cosmic Tools Limited, Coimbatore. Nine slots were made on each specimen at different intervals. The tests were conducted for surface roughness (Ra) and kerf width for the three specimens after carrying out the wire EDM machining using a brass wire of 0.25mm diameter. The deionized water was used as the dielectric. The details of the experimental and measurement set up were as follows:

- Machine: Wire EDM (Model:DK7750, M/s Concord United Products Private Limited, Bangalore, India);
- Electrode: Brass wire (0.25 mm diameter);
- Dielectric fluid: Deionized water;
- Flushing flow rate: 9 l/min;
- Surface roughness measuring device: Mitutoyo, Model: Surftest SJ-201 (Mitutoyo, Kanagawa, Japan); and
- Kerf width measuring device: Tool-makers’ microscope, Make: M/s Radical Scientific, Punjab, India.

Kerf width was measured using a tool-maker’s microscope with 100× magnification level used. The surface roughness measurement was carried out in the tester with 1 micron accuracy.

**Figure 4:**
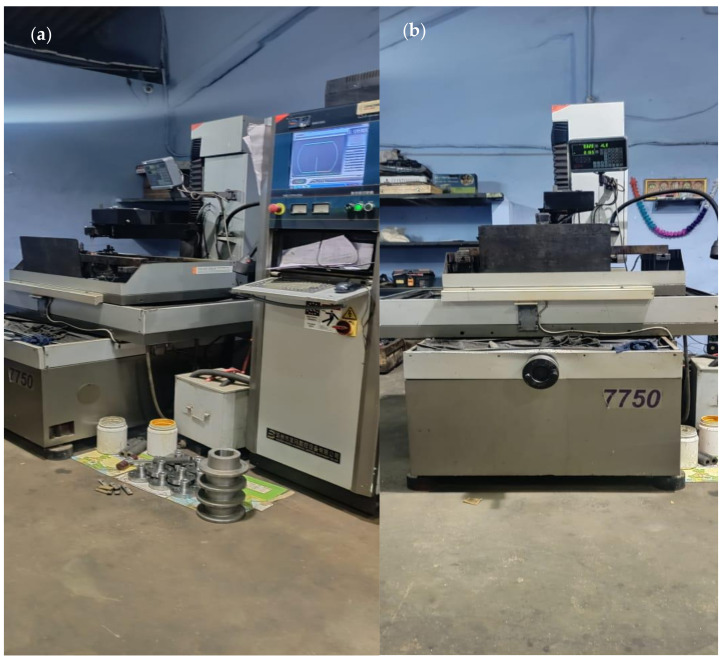
Wire EDM machine set up. (**a**) Isometric view, (**b**) Front view.

The experimental set up of the wire EDM is shown in Figure 4.

**Figure 5:**
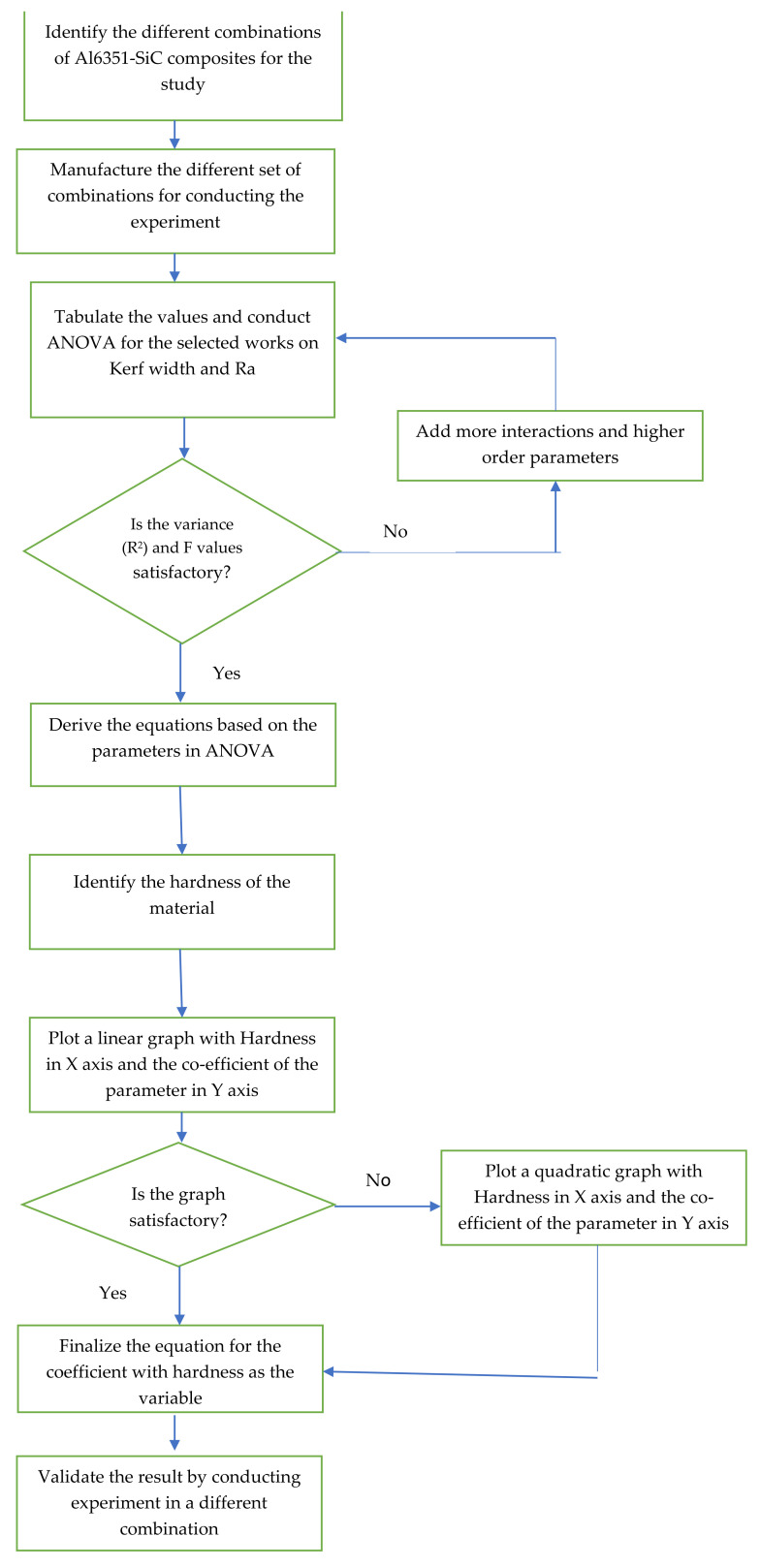
Flow chart of the methodology adopted.

The values were used to develop the mathematical models using Analysis of Variance (ANOVA) satisfying the requirements of variance (R^2^) level. The methodology adopted is provided as a flow chart in Figure 5.

The original works along with the experimental values were taken as the reference and the reduced ANOVA ignoring the less important factors were used for this model creation. The methodology adopted for carrying out the study is as follows:

- The mathematical equations were developed for the surface roughness and kerf width based on the ANOVA;
- The mathematical equations developed for different combinations were based on different parameters and were listed and compared for the materials under consideration;
- The variations observed were identified and the pattern of variation was observed based on the hardness for the composites;
- The coefficients of each parameter which are considered are compiled and a graph is generated with the parameter on the Y-axis and the hardness on the X-axis — this graph is also checked for its precision levels and the best matching plots are taken for further studies;
- This pattern was used for creating the equations for the coefficients of different parameters under consideration based on the hardness of the material and these values were used to predict the output parameters;
- The functional equation is validated by carrying out experiments for a selected combination of the Al 6351–SiC–B_4_C composite; and
- Based on the functional equations, for a new material combination of Al6351-based composites with different hardness, the surface roughness as well as kerf width can be predicted for the specified input parameters.

## 3. Results and Discussion

The work aimed at identifying an efficient and simple methodology to predict the kerf width and surface roughness of an Al6351–SiC composite with different percentages of B_4_C as the reinforcement. The analysis was taken from the machining of the composite with 0, 5, and 10% B_4_C. There was a total of 27 experimental results, which were classified into three categories of nine experiments each based on the percentage of B_4_C. Along with these three sets, three more samples were developed with 3, 6, and 8% of B_4_C added to Al6351–5% SiC. The addition of more B_4_C in mixture was found to be difficult in providing a proper mixing of the reinforcement in the matrix. Therefore, the sample developed with 12% B_4_C was discarded as the mixture has porosity identified on the surface. The input parameters taken for the study were current (A), pulse-on-time (T_on_), and wire feed rate (WFR). The output parameters were kerf width (KRW) and surface roughness (R_a_). Degree of freedom (DF), sum of squares (SS), contribution of each parameter, mean of squares (MS), and F-values.

Based on the experimental values, ANOVA was carried out and the details of the same has been provided in Table 1, Table 2, Table 3, Table 4, Table 5, Table 6, Table 7, Table 8, Table 9, Table 10, Table 11 and Table 12.

The corresponding equations obtained were:

KRW = 0.1013 − 0.01429A + 0.002878T_on_ − 0.01061WFR + 0.000177A × A + 0.000050A × T_on_+ 0.000562A × WFR(1)

R_a_ = −35.6 + 4.14A + 0.354T_on_ + 0.099WFR − 0.133A × A − 0.0398A × T_on_ + 0.0000A × WFR (2)

The corresponding equations obtained were:

KRW = 0.167 − 0.01333A − 0.001333 T_on_ − 0.00333WFR + 0.001250A × A + 0.000083A × T_on_ − 0.000208A × WFR (3)

R_a_ = −47.4 + 3.70A + 0.543T_on_ − 1.075WFR − 0.0178A × A − 0.04154A × T_on_ + 0.1737A × WFR(4)

The corresponding equations are:

KRW = −0.046 + 0.0018A + 0.00243T_on_ − 0.0010 WFR − 0.000375A × A + 0.000079A × T_on_ + 0.000271A × WFR (5)

R_a_ = −9.1 + 0.6A + 0.152T_on_ − 0.281 WFR − 0.023A × A − 0.0109A × T_on_ + 0.0129A × WFR (6)

The corresponding equations are:

KRW = 0.303 − 0.0558A + 0.00044T_on_ − 0.0128WFR + 0.002083A × A + 0.000167A × T_on_ + 0.000833A × WFR(7)

R_a_ = −20.0 + 2.17A + 0.187T_on_ − 0.096WFR − 0.0376A × A − 0.01637A × T_on_ + 0.0950A × WFR(8)

The corresponding equations are:

KRW = 0.0800 − 0.01750A + 0.000111T_on_ + 0.00722WFR + 0.001458A × A + 0.000083A × T_on_ − 0.000208A × WFR(9)

R_a_ = −25.0 + 2.79A + 0.310T_on_ − 1.035WFR − 0.0294A × A − 0.03021A × T_on_ + 0.1940A × WFR (10)

The corresponding equations are

KRW =0.0422 − 0.00983A + 0.003183T_on_ + 0.000292A × A − 0.000000A × T_on_ (11)

R_a_ = −17.7 + 2.14A + 0.025T_on_ − 0.0687A × A + 0.00088A × T_on_ (12)

ANOVA table clearly indicates that the parameters used are providing a clear idea for the output parameters except for the surface roughness of Al6351–5% SiC–10% B_4_C composite where the F values are slightly lower than the required range and the variance is 70.9%. Except for this, all the ANOVA shows satisfactory values of variance and the F-value. The ANOVA table tallies with the literature reviews carried out which provides the major contributor as current or pulse-on-time for the kerf width. It was also identified that it differs with some of the literatures where surface roughness was dominated by pulse-on-time. Our work identified that the wire feed rate is the major contributor which matched with some of the literature.

The material property considered for the evaluation was hardness. The hardness value for the composite with 0, 5, and 10% B_4_C was taken from Sureshkumar [1]. The hardness of the remaining composites was measured using the Vickers hardness test apparatus available at Amal Jyothi College of Engineering, Kottayam, Kerala, India. The properties of the various combinations of the Al6351–5% SiC were identified and tabulated in Table 13.

**Figure 6:**
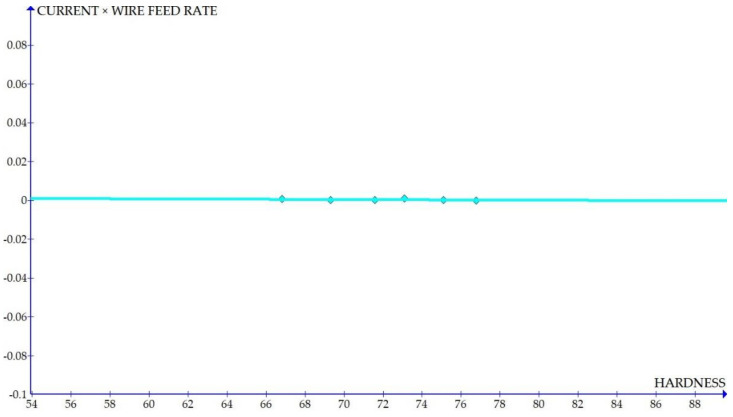
Coefficient of kerf width: current × WFR vs. hardness.

**Figure 7:**
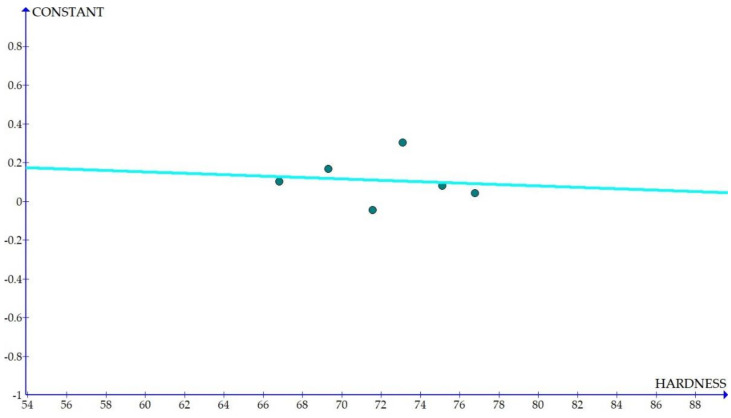
Coefficient of kerf width: constant vs. hardness.

**Figure 8:**
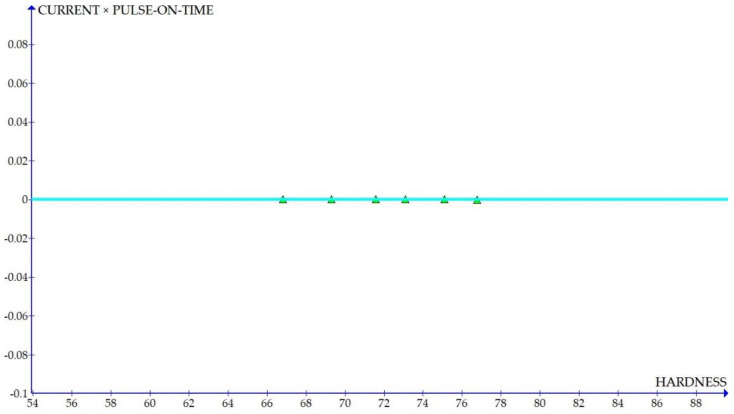
Coefficient of kerf width: current × pulse on time vs. hardness.

**Figure 9:**
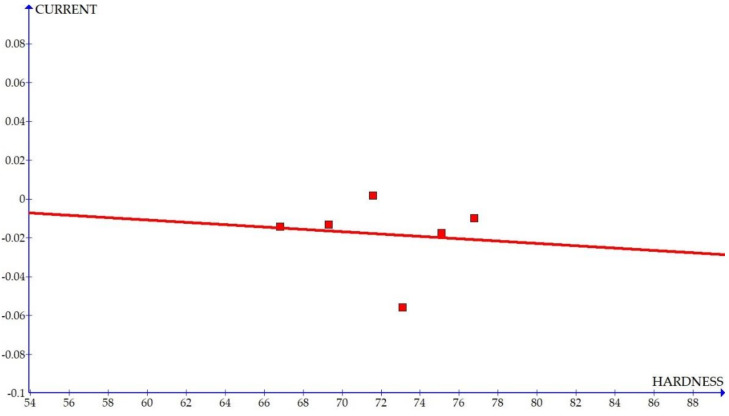
Coefficient of kerf width: current vs. hardness.

**Figure 10:**
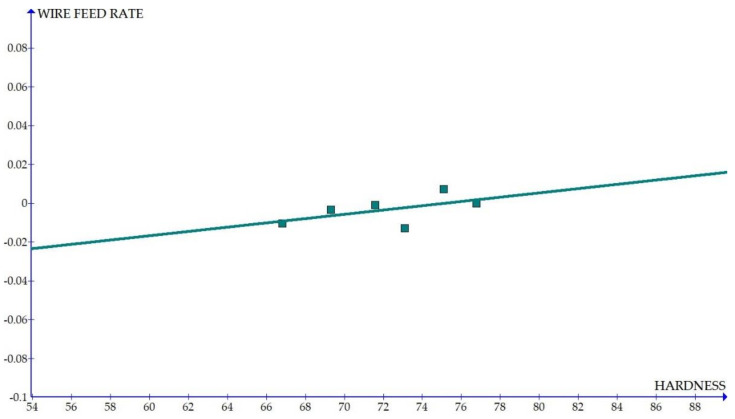
Coefficient of kerf width: wire feed rate vs. hardness.

**Figure 11:**
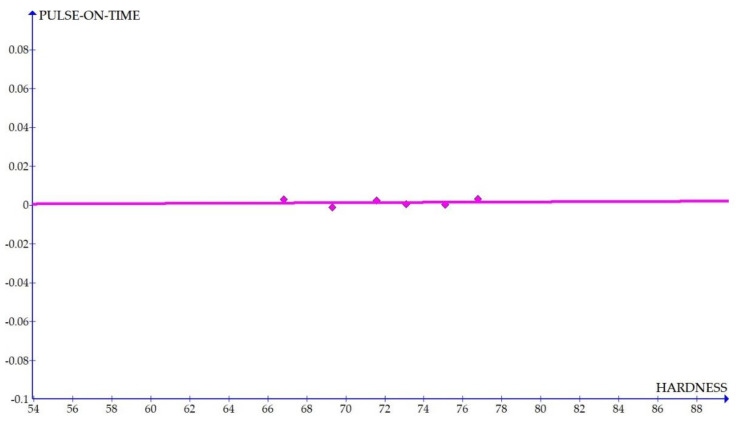
Coefficient of kerf width: pulse on time vs. hardness.

**Figure 12:**
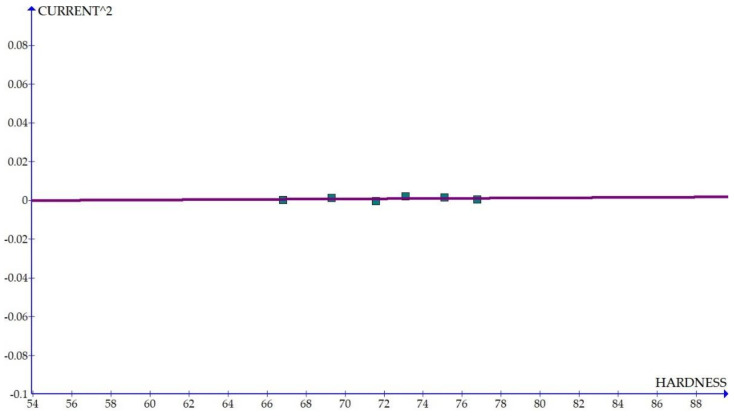
Coefficient of kerf width: current^2^ vs. hardness.

The variations of the different coefficients according to the variation in the hardness were plotted and the equations were obtained based on the hardness of the composite for different compositions. The details of the graph obtained are provided in Figure 6, Figure 7, Figure 8, Figure 9, Figure 10, Figure 11 and Figure 12 for the kerf width and in Figures figure the surface roughness. The graphs aimed at developing a simple methodology that can predict the output parameters with minimum errors.

Figure 6, Figure 7, Figure 8, Figure 9, Figure 10, Figure 11 and Figure 12 show the graph for variation of different coefficients of kerf width vs. hardness of the material. From the graphs, it can be understood that the parameters of current and constant shows a larger slope which indicate that these two parameters are the major contributors in the variation of the kerf width with respect to hardness. All the other considered parameters show a closer-to-zero slope which indicates its lesser influence on variation of kerf width due to the variation in the hardness of the composite.

**Figure 13:**
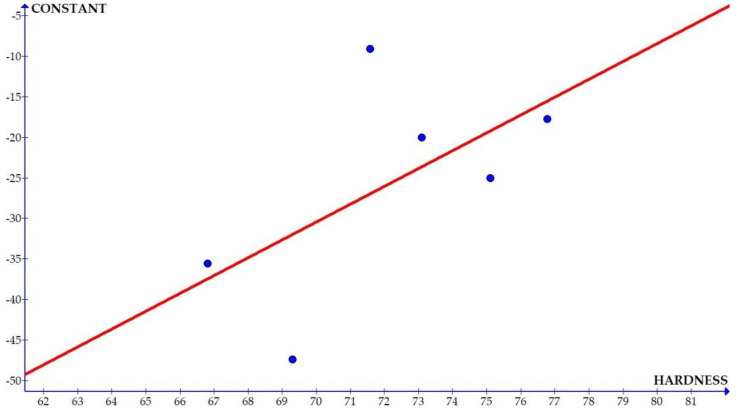
Coefficient of surface roughness: constant vs. hardness.

**Figure 14:**
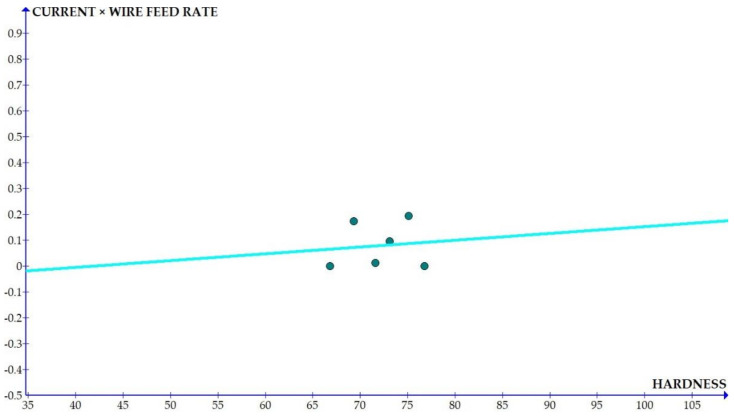
Coefficient of surface roughness: WFR × current vs. hardness.

**Figure 15:**
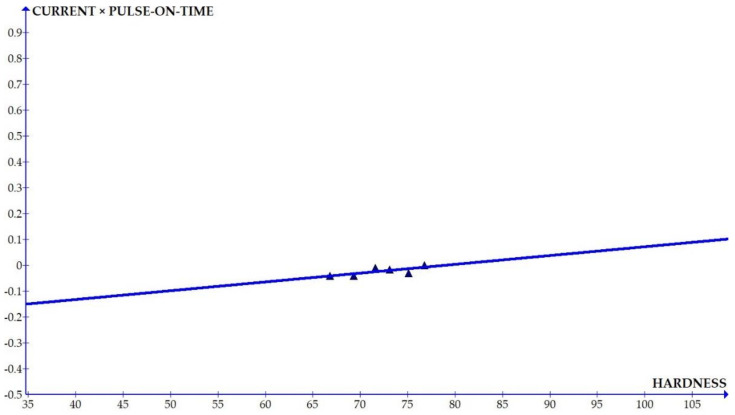
Coefficient of surface roughness: pulse on time × current vs. hardness.

**Figure 16:**
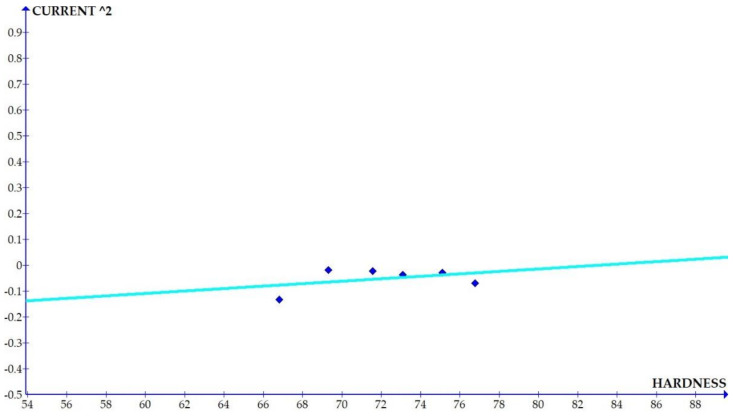
Coefficient of surface roughness: current^2^ vs. hardness.

**Figure 17:**
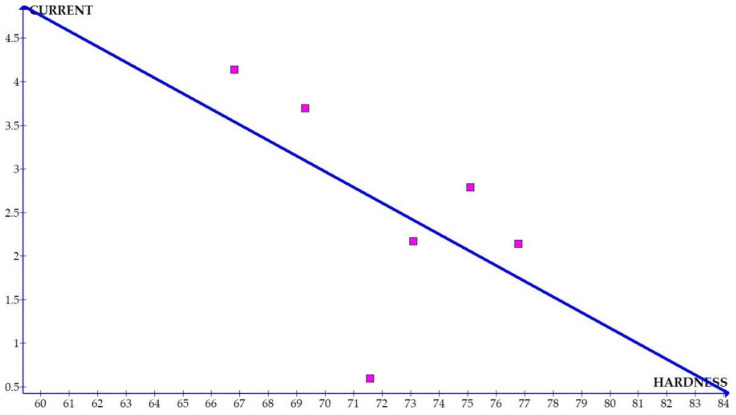
Coefficient of surface roughness: current vs. hardness.

**Figure 18:**
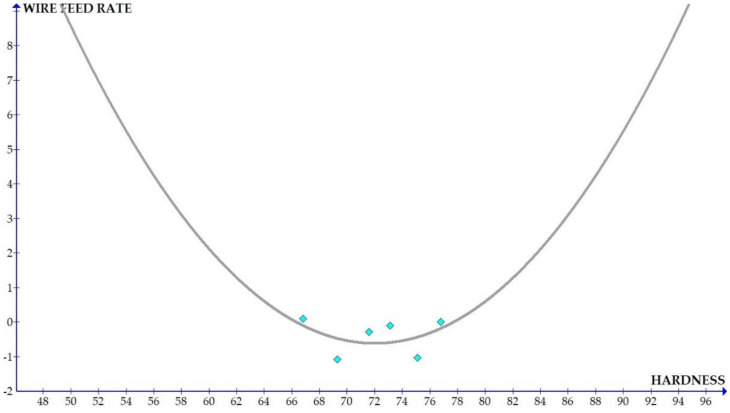
Coefficient of surface roughness: feed vs. hardness.

**Figure 19:**
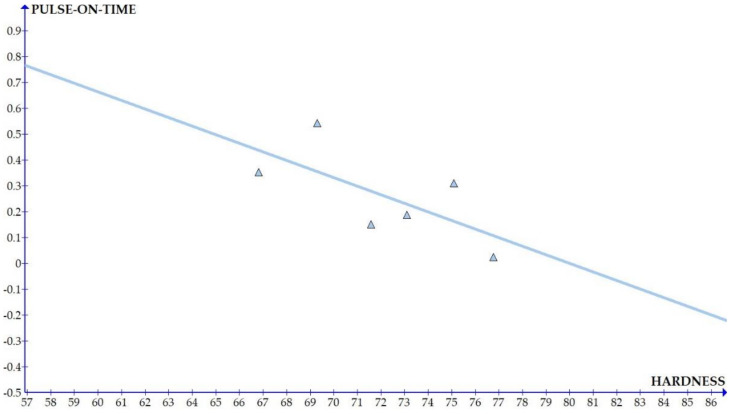
Coefficient of surface roughness: pulse on time vs. hardness.

Figure 13, Figure 14, Figure 15, Figure 16, Figure 17, Figure 18 and Figure 19 present details of the graph obtained for the surface roughness.

Figure 13, Figure 14, Figure 15, Figure 16, Figure 17, Figure 18 and Figure 19 show the variation in the coefficient of different parameters against hardness for the output parameter ‘surface roughness’. The coefficients of current and pulse on time shows a negative slope which indicates that the increase of these parameters diminishes the surface finish with increase in hardness. The curve of wire feed rate is a quadrilateral curve which is showing a very low slope at the hardness ranges related to the Al6351 composite. 

Based on the graphs, the coefficient Equations based on the Brinell hardness (HRB) (13–19) were obtained for the kerf width.

Constant = −0.0036 × HRB + 0.3695(13)

T_on_ = 4.1197 × 10^−5^ × HRB − 0.0017 (14)

WFR = 0.0011 × HRB − 0.083(15)

A =−0.0006 × HRB + 0.0254 (16)

A^2^ = 5.1845 × 10^−5^ × HRB − 0.0029 (17)

A × T_on_ = −1.8732 × 10^−6^ × HRB + 0.0002 (18)

Current × WFR =−3.329 × 10^−5^ × HRB + 0.0027 (19)

Based on the graphs, the coefficient Equations (20-26) were obtained for the surface roughness.

Constant = 2.2023 × HRB − 184.6096(20)

T_on_ =−0.0332 × HRB + 2.6548 (21)

WFR = 0.019 × HRB^2^ − 2.7303 × HRB + 97.6943 (22)

A =−0.1794 × HRB + 15.527(23)

A^2^ = 0.0047 × HRB − 0.3927 (24)

A × T_on_ = 0.0034 × HRB − 0.2682 (25)

A × WFR = 0.0026 × HRB − 0.1099(26)

The graph and the equations were generated using graph software and it was targeted to create the simplest method for the prediction. Therefore, linear variations were considered for maximum coefficients considering experimental errors and variation in ANOVA. The higher slope of the graph indicates larger variations or in other words, larger dependence on the hardness of the material. ANOVA can be employed to identify the most contributing factor, whereas equations of coefficients aim at identifying the factor that varies considerably due to variation in the material property considered, which is hardness. The graph had provided a clear indication on how each coefficient varies according to the hardness of the material. It was also observed that the fit value (R^2^) was satisfactory (above 0.6) for all the linear equations generated except for two equations. Therefore, these equations can be employed for the evaluation of the machining system. If we monitor the graphs, we can clearly see that most of the factors had a positive slope which implies that increase of hardness increases most of the factors pertaining to the kerf width and the surface roughness of the material. But the most contributing factor of constant had a negative slope in case of surface roughness which shows that the parameter should decrease with the increase in hardness.

The corresponding equations shows that the variation of the hardness creates a linear variation in most of the input parameters as well as interactions. The wire feed rate which is the most dominating parameter showed a second-degree variation which shows that the increase in the hardness reduces the surface quality drastically as we can see in the graph. Therefore, increased hardness calls for a lower WFR for obtaining good surface finish.

## 4. Validation

**Figure 20:**
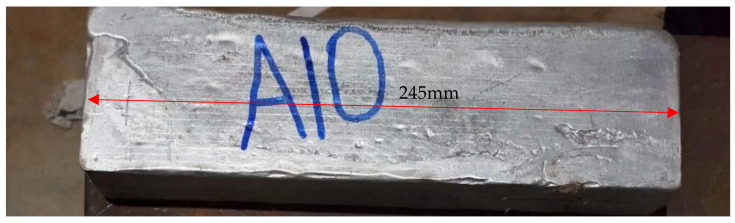
Fabricated sample.

The equations obtained were validated by carrying out experiments on Al6351–5% SiC–1% B_4_C. The composite was manufactured using stir casting at M/s Initially the casting die is preheated to 400 °C. Al6061 is cut to small pieces to fit into the crucible and is kept in the furnace. The base material was heated to preset temperature of 850 °C. The molten metal is stirred using a stirrer at 700 rpm and the reinforcement is slowly added to the metal. Magnesium in amount 2% is also added to the molten metal for better adherence of the metal and the reinforcement. The mixture is poured into the preheated die to obtain the final composite. The final cast composite is shown in Figure 20.

**Figure 21:**
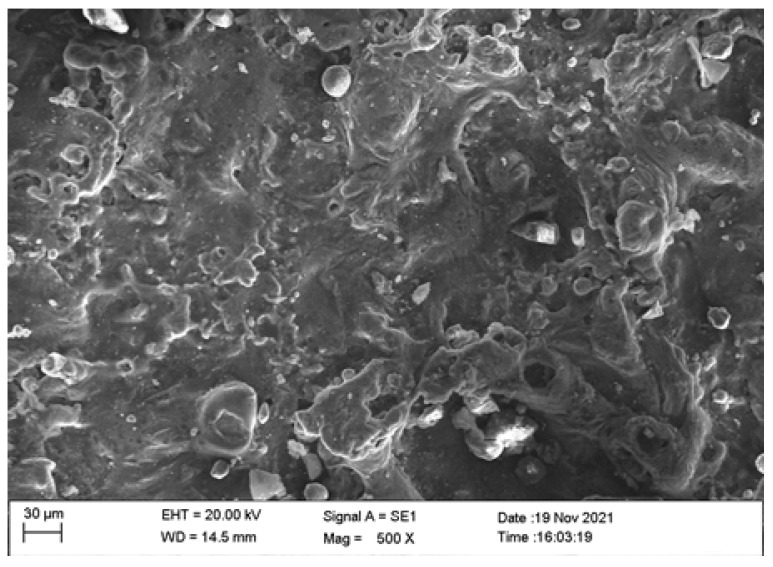
Morphology of machined surface.

SEM analysis was conducted on the machined surface of the specimen to confirm the proper distribution of the reinforcements in the base matrix. The SEM image obtained is provided in Figure 21. The SEM image clearly shows the even distribution of the reinforcements in the base matrix. The wire EDM machining on the surface of the machined component created a white layer on the machined surface, due to the intermittent application of electrical energy. During pulse-on-time the composite melts and then it cools during the pulse-off-time, creating a layer on the surface of the composite which is known as the white layer. It can also be observed the presence of micro craters on the machined surface of the specimen due to the wire EDM machining. These craters reduce the surface finish of the material.

**Figure 22:**
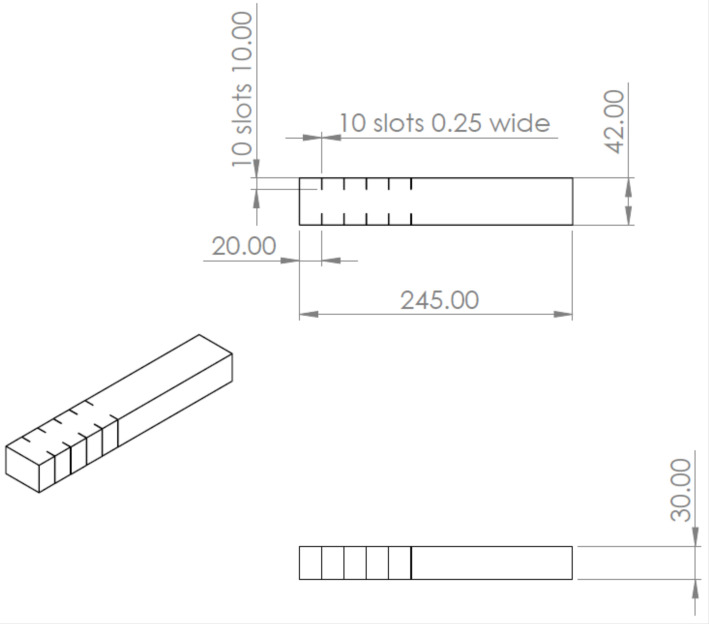
Drawing of the specimen with dimension for wire EDM cut.

Figure 22 provides the dimension of the workpiece and the details of the cut developed through wire EDM. The hardness of the composite was tested using Vickers hardness testing machine and was converted to HRB using standard table. The hardness was tested for a load of 10N with a dwell time of 10s. The values were taken at three points and the average was taken as the final hardness. The values observed at the three points were 67.6, 68.8, and 68.5. The final hardness of the specimen was identified as 68.3 HRB.

The experiment was carried out for different parameters. The details of the parameters are provided in Table 14.

Based on the hardness value obtained, the values of kerf width and surface roughness were determined using the co-efficient equations derived. The values are compared with the experimental results and the variation was tabulated in Table 15. 

The result of the experiment is showing a close relationship with the mathematical model. The maximum variation identified in the kerf width was 13.57% and that of the surface roughness was 20.89%. The variations observed can be attributed to the approximations in the graphs and the corresponding equations. More accurate equations with higher-order terms can improve the values but will complicate the mathematical model. Since the present mathematical model satisfies the experimental results with very low variations, these results can be used for predicting the kerf width and the surface roughness of the Al6351 composite with a different combination of reinforcements.

## 5. Conclusions

The result obtained through experiment was very close to the calculated values within 15% variation, except for two values in surface roughness. This method has used linear regression for the prediction except for WFR in surface roughness, which makes this methodology simpler. Therefore, the present model can be employed for the prediction of the kerf width and surface roughness of the any Al6351 based composites with the input as hardness of the developed composite. It can be noted that maximum variation observed was 13.57% and 20.89% for kerf width and surface roughness, respectively. Therefore, this simple method can provide an approximate value for the considered parameters. This method can be employed for any of the aluminum-based composite for wire-cut EDM as this method provides a simple and effective method for predicting the two major parameters concerning the wire-cut EDM process. It can be identified that the current and pulse-on-time are the major factors affecting the kerf width whereas the wire feed rate has been observed to be the major contributor to the surface roughness.

The work can be successfully used for prediction of the kerf width and surface roughness of Al6351–SiC–B_4_C composites as the maximum variations are within the limits. But further work needs to be carried out for extending the work for different combinations of reinforcements in Al6351 to convert this work into a generalized work. This method can be applied for different composites with different matrix materials for generating similar equations. The major disadvantage of this work is that the work was limited to similar composites as the behaviour of different composites were identified to be drastically varying for EDM and wire EDM based machining.

## Figures and Tables

**Table 1:** Analysis of variance: kerf width—Al6351–5% SiC–0% B_4_C.

Source	DF	SS	Contribution	MS	F-Value
Regression	6	0.007797	99.91%	0.001300	383.48
A	1	0.000181	2.33%	0.000181	53.56
T_on_	1	0.007420	95.08%	0.007420	2189.56
WFR	1	0.000047	0.60%	0.000047	13.79
A × A	1	0.000016	0.21%	0.000016	4.74
A × T_on_	1	0.000072	0.93%	0.000072	21.32
A × WFR	1	0.000061	0.78%	0.000061	17.93
Error	2	0.000007	0.09%	0.000003	-
Total	8	0.007804	100.00%	-	-

**Table 2:** Analysis of variance: surface roughness—Al6351–5% SiC–0% B_4_C.

Source	DF	SS	Contribution	MS	F-Value
Regression	6	2.87767	95.20%	0.47961	6.62
A	1	0.11788	3.90%	0.11788	1.63
T_on_	1	2.55845	84.64%	2.55845	35.29
WFR	1	0.17602	5.82%	0.17602	2.43
A × A	1	0.02115	0.70%	0.02115	0.29
A × T_on_	1	0.00416	0.14%	0.00416	0.06
A × WFR	1	0.00000	0.00%	0.00000	0.00
Error	2	0.14498	4.80%	0.07249	-
Total	8	3.02265	100.00%	-	-

**Table 3:** Analysis of variance: kerf width—Al6351–5% SiC–3% B_4_C.

Source	DF	SS	Contribution	MS	F-Value
Regression	6	0.113967	99.97%	0.018994	1139.67
A	1	0.112067	98.30%	0.112067	6724.00
T_on_	1	0.000267	0.23%	0.000267	16.00
WFR	1	0.000800	0.70%	0.000800	48.00
A × A	1	0.000800	0.70%	0.000800	48.00
A × T_on_	1	0.000025	0.02%	0.000025	1.50
A × WFR	1	0.000008	0.01%	0.000008	0.50
Error	2	0.000033	0.03%	0.000017	-
Total	8	0.114000	100.00%	-	-

**Table 4:** Analysis of variance: surface roughness—Al6351–5% SiC–3% B4C.

Source	DF	SS	Contribution	MS	F-Value
Regression	6	63.6102	99.21%	10.6017	41.70
A	1	0.2200	0.34%	0.2200	0.87
T_on_	1	1.4357	2.24%	1.4357	5.65
WFR	1	52.2617	81.51%	52.2617	205.58
A × A	1	0.1623	0.25%	0.1623	0.64
A × T_on_	1	3.7384	5.83%	3.7384	14.71
A × WFR	1	5.7921	9.03%	5.7921	22.78
Error	2	0.5084	0.79%	0.2542	-
Total	8	64.1187	-	-	-

**Table 5:** Analysis of variance: kerf width—Al6351–5% SiC–5% B_4_C.

Source	DF	SS	Contribution	MS	F-Value
Regression	6	0.010162	97.19%	0.001694	11.51
A	1	0.000043	0.41%	0.000043	0.29
T_on_	1	0.009761	93.35%	0.009761	66.32
WFR	1	0.000200	1.91%	0.000200	1.36
A × A	1	0.000072	0.69%	0.000072	0.49
A × T_on_	1	0.000072	0.69%	0.000072	0.49
A × WFR	1	0.000014	0.13%	0.000014	0.10
Error	2	0.000294	2.81%	0.000147	-
Total	8	0.010456	100%	-	-

**Table 6:** Analysis of variance: surface roughness—Al6351–5% SiC–5% B_4_C.

Source	DF	SS	Contribution	MS	F-Value
Regression	6	2.54846	88.59%	0.42474	2.59
A	1	0.01520	0.53%	0.01520	0.09
T_on_	1	2.28043	79.27%	2.28043	13.89
WFR	1	0.10260	3.57%	0.10260	0.63
A × A	1	0.10672	3.71%	0.10672	0.65
A × T_on_	1	0.01177	0.41%	0.01177	0.07
A × WFR	1	0.03172	1.10%	0.03172	0.19
Error	2	0.32833	11.41%	0.16416	-
Total	8	2.87678	100%	-	-

**Table 7:** Analysis of variance: kerf width—Al6351–5% SiC–6% B4C.

Source	DF	SS	Contribution	MS	F-Value
Regression	6	0.132044	99.76%	0.022007	141.48
A	1	0.123267	93.13%	0.123267	792.43
T_on_	1	0.006017	4.55%	0.006017	38.68
WFR	1	0.000006	0.00%	0.000006	0.04
A × A	1	0.002222	1.68%	0.002222	14.29
A × T_on_	1	0.000400	0.30%	0.000400	2.57
A × WFR	1	0.000133	0.10%	0.000133	0.86
Error	2	0.000311	0.24%	0.000156	-
Total	8	0.132356	100.00%	-	-

**Table 8:** Analysis of variance: surface roughness—Al6351–5% SiC–6% B_4_C.

Source	DF	SS	Contribution	MS	F-Value
Regression	6	42.6097	98.77%	7.1016	26.77
A	1	0.5953	1.38%	0.5953	2.24
T_on_	1	2.7608	6.40%	2.7608	10.41
WFR	1	36.4943	84.59%	36.4943	137.57
A × A	1	0.7240	1.68%	0.7240	2.73
A × T_on_	1	0.3025	0.70%	0.3025	1.14
A × WFR	1	1.7328	4.02%	1.7328	6.53
Error	2	0.5305	1.23%	0.2653	-
Total	8	43.1403	100.00%	-	-

**Table 9:** Analysis of variance: kerf width—Al6351–5% SiC–8% B_4_C.

Source	DF	SS	Contribution	MS	F-Value
Regression	6	0.132478	99.99%	0.022080	3974.33
A	1	0.129067	97.42%	0.129067	23,232.00
T_on_	1	0.002017	1.52%	0.002017	363.00
WFR	1	0.000272	0.21%	0.000272	49.00
A × A	1	0.001089	0.82%	0.001089	196.00
A × T_on_	1	0.000025	0.02%	0.000025	4.50
A × WFR	1	0.000008	0.01%	0.000008	1.50
Error	2	0.000011	0.01%	0.000006	-
Total	8	0.132489	100.00%	-	-

**Table 10:** Analysis of variance: surface roughness—Al6351–5% SiC–10% B_4_C.

Source	DF	SS	Contribution	MS	F-Value
Regression	6	86.7177	99.04%	14.4529	34.38
A	1	0.6273	0.72%	0.6273	1.49
T_on_	1	0.6734	0.77%	0.6734	1.60
WFR	1	77.0040	87.95%	77.0040	183.19
A × A	1	0.4418	0.50%	0.4418	1.05
A × T_on_	1	0.7482	0.85%	0.7482	1.78
A × WFR	1	7.2230	8.25%	7.2230	17.18
Error	2	0.8407	0.96%	0.4204	-
Total	8	87.5584	100.00%	-	-

**Table 11:** Analysis of variance: kerf width—Al6351–5% SiC–10% B_4_C.

Source	DF	SS	Contribution	MS	F-Value
Regression	4	0.006148	99.45%	0.001537	181.71
A	1	0.000024	0.39%	0.000024	2.84
T_on_	1	0.006080	98.36%	0.006080	718.84
A × A	1	0.000044	0.70%	0.000044	5.15
A × T_on_	1	0.000000	0.00%	0.000000	0.00
Error	4	0.000034	0.55%	0.000008	-
Total	8	0.006182	100.00%	-	-

**Table 12:** Analysis of Variance: Surface roughness—Al6351–5%SiC–10%B_4_C.

Source	DF	SS	Contribution	MS	F-Value
Regression	4	3.50599	70.91%	0.87650	2.44
A	1	0.16170	3.27%	0.16170	0.45
T_on_	1	0.92591	18.73%	0.92591	2.57
A × A	1	2.41340	48.81%	2.41340	6.71
A × T_on_	1	0.00497	0.10%	0.00497	0.01
Error	4	1.43852	29.09%	0.35963	-
Total	8	4.94451	100.00%	-	-

**Table 13:** Material composition of Al6351–5% SiC composite with different percentages of B_4_C.

Composites	Yield Strength (N/mm^2^)	Tensile Strength (N/mm^2^)	Density (kg/m^3^)	Hardness (HB)
Al–5 wt.% SiC Al–5 wt.% SiC–3 wt.% B_4_C Al–5 wt.% SiC–5 wt.% B_4_C Al–5 wt.% SiC–6 wt.% B_4_C Al–5 wt.% SiC–8 wt.% B_4_C Al–5 wt.% SiC–10 wt.% B_4_C	81.37 84.54 98.75 100.27 105.41 107.43	105.62 109.12 120.32 124.36 129.12 132.48	2725 2720 2715 2712 2707 2705	66.81 69.3 71.58 73.1 75.1 76.78

**Table 14:** Process Parameters used in the experiments.

Parameters	Values
Current [A]	12, 16, and 20
Pulse-on-time [µm]	100, 110, and 120
Wire feed rate [m/min]	6, 8, and 10

**Table 15:** Validation table for Al6351–5% SiC–1% B_4_C.

A	T_on_	WFR	Kerf width (mm)			Surface roughness (Ra) (µm)		
			Calculated	Experimental	% Variation	Calculated	Experimental	% Variation
12	100	6	0.43158	0.38	−13.57	12.2021	11.1000	−9.929
12	110	10	0.44035	0.4	−10.09	18.5572	15.3500	−20.894
12	120	8	0.46564	0.46	−1.23	17.1707	16.9500	−1.302
16	100	6	0.48011	0.43	−11.65	8.5390	7.6500	−11.620
16	110	10	0.49858	0.46	−8.39	15.3028	14.0200	−9.150
16	120	8	0.52334	0.5	−4.67	12.3727	10.6800	−15.849
20	100	6	0.54915	0.51	−7.68	4.0806	3.5500	−14.946
20	110	10	0.57732	0.54	−6.91	9.2531	8.9000	−3.967
20	120	8	0.60156	0.55	−9.37	4.7795	4.2000	−13.797
